# Navigated Transcranial Magnetic Stimulation Motor Mapping and Diffusion Tensor Imaging Tractography for Diencephalic Tumor in Pediatric Patients

**DOI:** 10.3390/brainsci13020234

**Published:** 2023-01-30

**Authors:** Valentina Baro, Luca Sartori, Samuel Luciano Caliri, Giulia Melinda Furlanis, Alberto D’Amico, Giulia Meneghini, Silvia Facchini, Florinda Ferreri, Maurizio Corbetta, Luca Denaro, Andrea Landi

**Affiliations:** 1 Pediatric and Functional Neurosurgery, Department of Neuroscience, University of Padova, 35128 Padova, Italy; 2 Padova Neuroscience Center (PNC), University of Padova, 35128 Padova, Italy; 3 Department of Neuroscience, University of Padova, 35128 Padova, Italy; 4 Unit of Neurology, Unit of Clinical Neurophysiology and Study Center for Neurodegeneration (CESNE), Department of Neuroscience, University of Padua, 35128 Padua, Italy; 5 Department of Clinical Neurophysiology, Kuopio University Hospital, University of Eastern Finland, 70211 Kuopio, Finland; 6 Venetian Institute of Molecular Medicine (VIMM), 35128 Padova, Italy

**Keywords:** thalamopeduncolar tumor, thalamic tumor, children, nTMS, corticospinal tract

## Abstract

*Background.* In deep-seated brain tumors, adequate preoperative planning is mandatory to assess the best surgical corridor to obtain maximal safe resection. Functional diffusor tensor imaging (DTI) tractography based on navigated transcranial magnetic stimulation (nTMS) motor mapping has proven to be a valid preoperative examination method in adults. The aim of this paper is to present the application of nTMS and functional DTI tractography in a series of pediatric diencephalic tumors. *Material and methods.* Three patients affected by thalamic (one) and thalamopeduncular tumor (two) were successfully examined with nTMS motor mapping and DTI tractography between October 2020 and October 2021 (F:M 3:0, mean age 12 years ± 0.8). Cortical representation of leg, hand and mouth were determined in the affected hemisphere and the positive stimulation spots were set as seeds point for tractography. *Results.* Mapping of the motor cortex and tracts reconstruction for leg and hand were successful in all patients, while facial function was properly mapped in one patient only. In all cases, the procedure was well tolerated and no adverse events were recorded. Spatial relationships between tumor and functional tissue guided the surgical planning. Extent of the resection varied from 96.1% to 100% with a postoperative new motor deficit in one patient. *Conclusions.* nTMS and DTI fiber tracking is a feasible, effective and well-tolerated method to identify motor pathway in deep-seated lesion in pediatric population.

## 1. Introduction

In the last decade, the diffusion of navigated transcranial magnetic stimulation (nTMS) in neurosurgical practice has led to the consolidation of its precious role in preoperative motor and language mapping, especially in adult brain tumor surgery. The current literature defines nTMS as a non-invasive, well tolerated, safe and reliable method which is extremely helpful in presurgical planning [1,2,3,4,5,6,7,8,9,10,11,12,13,14]. Moreover, nTMS allows accurate functionally oriented white matter tracts reconstruction, also enabling sub-fascicle identifications. Thus, it facilitates comprehensive presurgical planning, particularly for subcortical and deep-seated lesions, in patients who are not eligible for awake surgery or in whom a direct cortical stimulation may facilitate the occurrence of intraoperative seizure [15,16,17,18,19,20,21,22,23].

Nevertheless, based on this large body of evidence, pediatric patients studied with nTMS have been rarely reported. Few papers have explored the application of nTMS to assess the cortical representation in epileptic children [8,24,25,26] and in preoperative evaluation for brain tumor surgery [27,28,29]. The epidemiology of central nervous system tumors favors the supratentorial localization after the age of 10 [30], providing a potentially collaborative population of patients that could benefit from nTMS cortical mapping. Moreover, older children overcome the intrinsic limitation related to the myelinization process and the need for a higher stimulation intensity. Even though younger or non-collaborative children cannot undergo speech assessment, cortical motor mapping could be passively realized under general anesthesia or sedation, with specific teams and settings [28].

Despite these drawbacks, nTMS could represent a valid alternative to functional MRI (fMRI), which is challenging to perform in sedated younger patients, due to its paradigm [31,32]. Since fMRI identifies areas of cortical activation by detecting the variations of blood oxygenation secondary to specific tasks, the depth of sedation and the type of sedative drug can interfere with both neuronal response and hemodynamic coupling, influencing image analysis and interpretation [33,34,35,36]. Focusing on the motor task, passive motion of lower extremities is less successful than that of upper extremities [32,33] and a young age has been found to limit the success of passive mapping [33]. Beyond that, it can be difficult to access a sedated patient positioned into the scanner because of the presence of intravenous lines and/or monitoring devices [32].

In this paper, a series of adolescent patients affected by diencephalic tumor who successfully underwent preoperative nTMS cortical mapping and diffusion tensor imaging (DTI) tractography for corticospinal tract (CST) identification is presented. The aim is to explore the feasibility and accuracy of the presurgical evaluation of subcortical pediatric lesions.

## 2. Material and Methods

### 2.1. Patient Selection

In this prospective observational study conducted between October 2020 and October 2021 at the Pediatric and Functional Neurosurgery, Department of Neuroscience, University of Padova, pediatric patients with supratentorial deep-seated lesions scheduled for surgical resection were screened. The main criteria for inclusion were (1) the potential benefit from motor subcortical tracts identification by means of nTMS-based DTI tractography, (2) the potential patient’s ability to collaborate during the nTMS cortical stimulation, (3) the eligibility of the patient for elective surgery.

Recorded patient data included *preoperative data*, i.e., demographic data, clinical presentation, length of symptoms, preoperative antiseizure medications, radiological data concerning tumor characteristics; *nTMS data*, i.e., adverse events, data regarding nTMS cortical mapping, resting motor threshold (RMT), number of stimuli, number of stimuli that elicited a motor evoked potential (MEP), identification of motor area for head/leg/face and subsequent *DTI tractography for CST*, identification and localization of CST bundles, fractional anisotropy (FA), tumor tract distance (DTT), fiber integrity; *intraoperative data*, i.e., surgical approach, intraoperative neuromonitoring (IONM) information, intraoperative complications, pathology; and *postoperative data*, i.e., postoperative complications, residual tumor volume, postoperative clinical status, clinical status at follow-up, need for complementary oncological treatment, length of follow-up.

The manuscript was written according to the Strengthening the Reporting of Observational Studies in Epidemiology (STROBE) checklist [37].

### 2.2. Patient Informed Consent and Ethical Approval

The patients’ parents signed specific informed consent for MRI acquisition, nTMS tests, neuropsychological evaluation and surgical intervention. The study was conducted in accordance with the ethical standards of the Institutional Research Committee AOUP (Prot. n 0001997 14 January 2021) and the 1964 Declaration of Helsinki, plus later amendments.

### 2.3. MRI Acquisition

The patient underwent brain MRI according to a specific protocol designed for nTMS and DTI tractography using a 3T scanner (Ingenia 3T, Philips Healthcare) to obtain 3D T1- weighted images (TR/repetition time = 8, TE/echo time = 3.7); 3D FLAIR/fluid attenuated inversion recovery (TR = 4800, TE = 299, TI/inversion time = 1650, flip angle = 40, matrix = 240 × 240 mm^2^, voxel = 1 × 1 × 1 mm^3^, 196 slices, 4.05 min of acquisition time); diffusion weighted sequences (DWI with 32 directions, TR = 4400, TE = 96; single shell, b = 800 s/mm^2^; multiband, 4.55 min of acquisition time) for DTI-FT. Diffusion weighted protocol was optimized compared to our previous report [23].

### 2.4. nTMS Motor Mapping

nTMS motor mapping was performed according to the most updated indications [12] using a figure-eight coil to stimulate the cortical area (NBS system 4.3—Nexstim Oy, Elimäenkatu 9 B, Helsinki, Finland). Recorded muscles were the first dorsal interosseous muscle and the abductor pollicis brevis for upper limb, the tibialis anterior and the abductor hallucis for lower limb, and the orbicularis oris for facial function. MEPs were recorded with the nTMS integrated electromyography using surface electrodes (NTMS EMG; sampling rate: 3 kHz/channel; analysis time: 10 ms pre-stimulus and 100 ms post-stimulus; filter bandwidth: 10–250 Hz). The determination of the RMT was performed according to previous reports [4,38].

### 2.5. Tractography

The workflow for DTI tractography was performed using a commercial software package (Elements, Brainlab, Munich, Germany). All positive spots were imported into the DTI software and enlarged to a diameter of 3 mm in order to create a continuous seed point area. The first region of interest (ROI) and a second ROI were set in the anteroinferior pons where the conventional color-coded fractional anisotropy (FA) map identified the CST. The FA was determined for each ROI and the fiber tracking was performed at 75% of FA using an anterograde direction with a vector step length of 1.6 mm, an angular threshold of 30° and a minimum length of 110 mm [20,21,22,39]. Fiber integrity was classified according to radiological appearance as (1) normal, (2) displaced but intact, (3) deformed, (4) interrupted [22].

## 3. Results

### 3.1. Patient Sample

**Figure 1:**
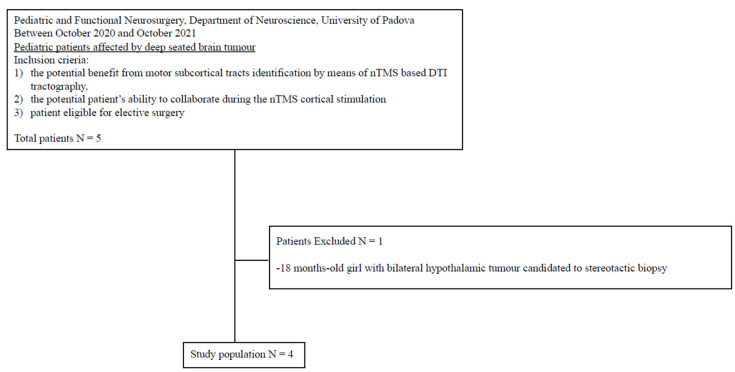
Flow diagram of patient selection.

During the enrollment, 20 pediatric patients were admitted for brain tumor. Only 5 patients out of 20 were affected by deep-seated lesion. After the application of inclusion criteria, four of them were scheduled for preoperative motor nTMS mapping. A complete flow diagram of patient selection is provided in Figure 1.

Three patients were female (M:F 1:3) and the mean age was 11.2 year ± 1.5. All of them had a diencephalic lesion: in one case purely thalamic, in two cases thalamopeduncular, and the only boy had a thalamic lesion with temporo-mesial and frontal invasion. One patient presented contralateral motor and VII cranial nerve deficits associated with a concomitant hydrocephalus (patient n. 2), while the other patients suffered from headache and episodes of vomiting. Preoperative data are reported in Table 1.

### 3.2. nTMS and Tractography

nTMS was performed successfully in three patients out of four (75%, F:M 3:0, mean age 12 years ± 0.8), while in one case (patient n. 4) the patient was extremely anxious, with oppositional behavior during the determination of RMT, despite the presence of the mother and the neuropsychologist, causing the permanent interruption of the session. In the other cases, cortical mapping was performed in one session, without interruption and without parental support. Moreover, no adverse events were recorded. All data regarding nTMS session are reported in Table 2, providing detailed information for upper and lower limb and for facial function. Only in one case (patient n. 3) was the cortical area for mouth successfully identified. Mapping was performed using a stimulation intensity of 110% RMT in all patients (approximately 80–100 V/m). The average duration of the whole session was 76.7 ± 19.4 min.

**Figure 2:**
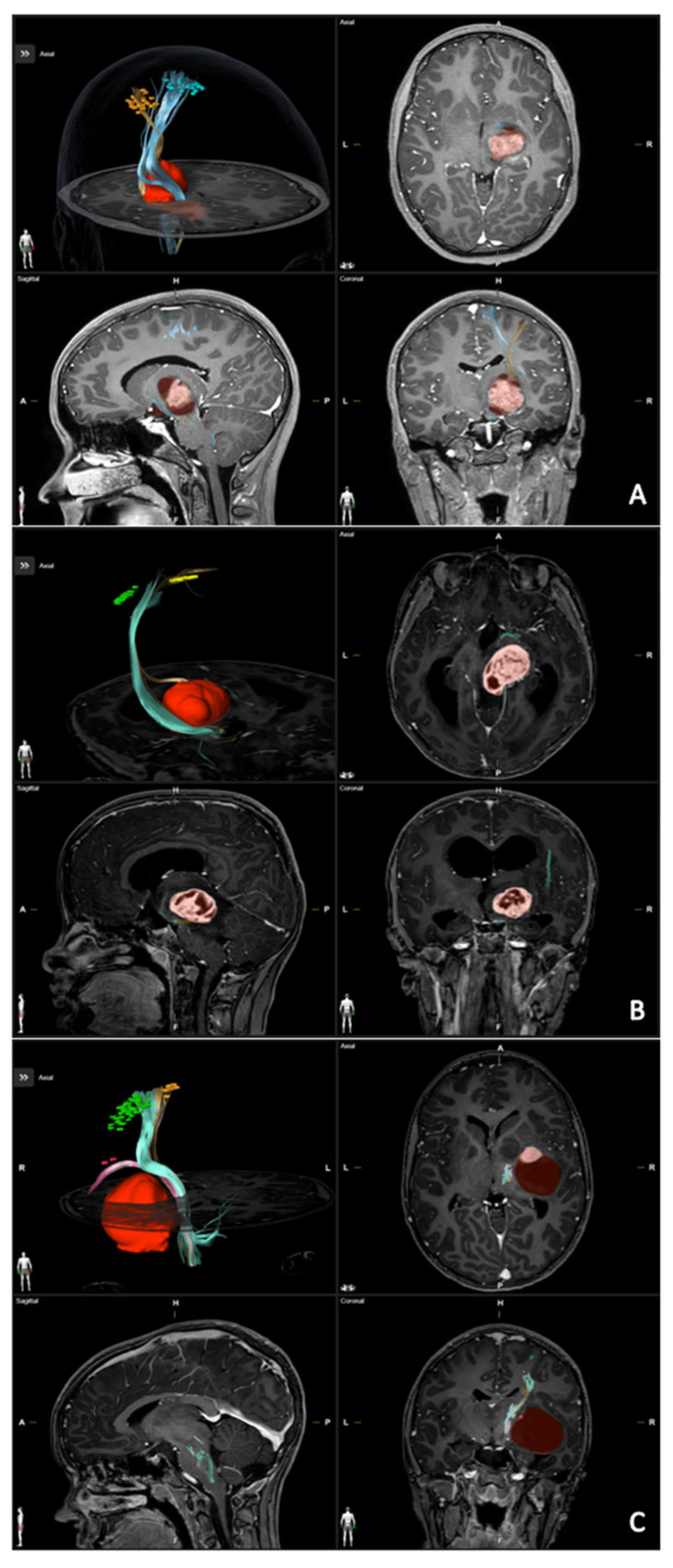
(**A**) Patient n. 1: right thalamopeduncular glioma causing a lateral displacement of CST with hand bundle splayed anteriorly (orange) and bundle for leg posteriorly. (**B**) Patient n. 2: right thalamopeduncular glioma with secondary hydrocephalus causing anterior displacement of the hand bundle (yellow) and the leg bundle posterolaterally. (**C**) Patient n. 3: right thalamic glioma leading to a medial displacement of the CST with bundle for mouth markedly shifted anteriorly (violet); hand (green) and leg (yellow) bundles medially.

In all cases, the CST was visualized, enabling the identification of the sub-bundles for hand and foot, while the sub-bundle for mouth was reconstructed only for patient n. 3. All CSTs were displaced but radiologically intact. The detailed localization of CST bundles are reported in Table 2. The distance between the lesion and the motor tract was ≤4 mm in all the patients. The results of the tractography were integrated into the surgical planning software, and the spatial relationships between CST sub-bundles were carefully evaluated, guiding the choice of more appropriate surgical approaches. All data are presented in detail in Table 2 and in Figure 2.

### 3.3. Surgery and Outcome

The surgical approach adopted was trans-sylvian in one case and trans-temporal trans-ventricular in all the others. All patients underwent surgery with IONM control and the white matter stimulation during tumor removal confirmed the results of tractography i.e., the sub-fascicles position and depth. In patient n. 1, at the end of resection, MEP were highly instable and the postoperative MRI disclosed a pericavitary ischemic lesion, resulting in a new motor deficit requiring intensive rehabilitation. Patient n. 2 had an important reduction of MEP during the resection stage in the infero-mesial part of the tumor, leading to a transient worsening of the pre-existing paresis in the postoperative period. All tumors were pilocytic astrocytoma; the extent of resection varied from 96.1% to 100% and the patients underwent clinical-radiological follow-up without adjuvant treatment. At the last follow-up, the patient with the postoperative new motor deficit was almost intact, and the patient with a worsened paresis was neurologically improved, presenting a better motor function compared with preoperative status. All data are presented in detail in Table 3.

## 4. Discussion

Despite the diffusion of nTMS motor and language mapping for preoperative surgical planning, this technique seems to be restricted to the adult population with brain tumors, with limited reports of pediatric patients [8,24,25,26,27,28,29]. The present study was conducted to investigate the application of nTMS motor mapping combined with DTI fiber tracking of the CST in pediatric deep-seated lesions, obtaining: (a) a successful mapping in the 75% of the patients; (b) the absence of adverse events; (c) a reliable identification of CST and relative sub-bundles, confirmed by IONM, guiding the choice of the more appropriate surgical corridor.

Several drawbacks limit the use of nTMS for cortical mapping in children, i.e., the immaturity of axon myelinization, the compliance of younger children or those with behavioral changes or neurodevelopment impairment, and the risk of nTMS-induced epileptic seizures. Coburger et al. reports a successful motor mapping performed in a partially awake 3-year-old boy, using higher stimulation intensities compared to those used in adults, without adverse events [28]. Mapping young children, especially under the age of 3, may lead to a lower rate of neuronal response due to the still ongoing myelinization process. Further, Schramm et al. found that mapping success was significantly associated with higher age; this result may be due to the above-mentioned insulation process of the CST in younger children but also to behavioral problems of the patients (both younger and older) hindering the reliability of the mapping [26]. Moreover, the risk of nTMS-induced seizures represents a deterrent to perform this mapping technique in children, due to rare cases detailing this adverse event, even with single-pulse stimulation in non-epileptic patients [40,41,42,43]. Nevertheless, studies on preoperative motor and language mapping in epileptic pediatric and adult patients have been reported without seizures during stimulation [8,24,25,26]. The rate of successful mapping in our series is 75%, slightly higher than previous reports, probably due to the higher age range of our patients [26,27].

In rolandic lesions, the identification of motor area and white matter tracts is crucial for surgical planning and individual risk stratification, becoming standard in clinical practice. Traditionally, DTI is obtained by diffusion weighted images through a deterministic algorithm with predefined anatomical landmarks used as ROIs and custom FA values. Unfortunately, this paradigm can be vitiated by the presence of surrounding oedema, tumor compression and/or distortion and by the proximity of bony structures. Specifically, in subcortical lesions the reconstruction of the tracts can be affected by the distortion and/or invasion caused by the tumor or by the presence of crossing bundles [44]. These well-known drawbacks may be overcome optimizing the MRI acquisition protocol and applying a probabilistic algorithm with dedicated post-processing teamwork to improve accuracy [45,46]. nTMS-based DTI tractography is functionally oriented, giving a more precise, reliable and accurate white matter reconstruction. In addition, this technique specifically identifies the sub-bundles of the major tracts, allowing a topographic knowledge of the fibers [3,20,21,22,27,39,47]. Nonetheless, the reconstruction can be performed directly by a neurosurgical team using commercial navigation software and the results can be easily used during surgery, saving time and resources. In our small cohort of patients, the accuracy of CST reconstruction was confirmed by IONM and in only one case was the bundle for facial function reconstructed. Indeed, the orbicularis oris only reacted to cortical stimulation, resulting in a valid MEP, in patient n. 3. As described by Schramm et al., during the stimulation of the area for the orbicularis oculi, the mapping may be hindered by artifacts due to direct nerve stimulation, undermining the mapping of such a small cortical area [26].

Preoperative risk stratification and postoperative deficit predictions based on nTMS results rely on RMT values, FA and DTT. A negative correlation between cortical excitability (higher RMT values) and tract integrity (lower FA) is associated with both pre and postoperative motor deficit. Furthermore, a DTT < 8 mm identifies a high-risk case for developing a new motor deficit after surgery [3,4,21,22]. From our data we can observe that in patient n. 3, RMT values were lower and FA higher compared to patient n. 2, who presented hemiparesis at diagnosis and who had a postoperative worsening of motor function. Unfortunately, due to the size of the sample, we cannot advance further speculations. In all cases the CST was displaced but radiologically intact and very close to the tumor, classifying the patients as high-risk for a new or worsened postoperative deficit. There is no doubt that CST identification by nTMS-based DTI tractography is incomparably superior to the standard MRI-derived DTI tractography, allowing a surgeon to choose an optimized and functionally guided surgical approach. Moreover, it enables a more precise preoperative risk assessment for postoperative motor deficits, improving preoperative counseling.

## 5. Limitations

Although there is homogeneity of the underlying pathology and demographic data in general, our sample size is small, prohibiting statistical analysis and generalizability of our results.

## 6. Conclusions

Preoperative nTMS motor mapping combined with nTMS-based DTI fiber tracking of the CST was feasible in the 75% of the patients without adverse events. The CST sub-bundles identification, their displacement and relationship with the tumor provide valuable information about the lesion and the surrounding eloquent areas, enabling a specific and more accurate surgical planning. Indeed, this information could help the decisions surrounding surgical approach, the surgical corridor and the extent of resection, in order to protect brain function. Furthermore, this method provides preoperative risk stratification, allowing a more detailed parent’s counselling.

## Figures and Tables

**Table 1:** Preoperative data.

	Patient 1	Patient 2	Patient 3	Patient 4
Preoperative Data				
Sex	F	F	F	M
Age	12	13	11	9
Tumor location	Right thalamopeduncular	Right thalamopeduncular	Right thalamic	Left thalamic with temporomesial and frontal invasion
Tumor volume (cm^3^)	12.3	19.0	36.7	42.3
Clinical Presentation	Headache and vomiting	Left hemiparesis and VII deficit, hydrocephalus	Headache and vomiting	Headache and vomiting
Motor status MRC grade (UL/IL)	M5/M5	M3/M3	M5/M5	M5/M5
Length of Symptoms (months)	12	7	3	0.2
Antiseizure medications	None	None	None	None
Other	-	NF1	NF1	-

F: female, M: male, MRC: motor research council, UL: upper limb, IL: inferior limb, NF1: neurofibromatosis type 1. See all data about patients in Table S1.

**Table 2:** nTMS and DTI tractography data.

**nTMS Data**			
Duration of the session (min)	73	102	55
Adverse event	None	None	None
RMT upper limb	43%	46%	35%
RMT lower limb	58%	60%	58%
RMT mouth	Not detected	Not detected	45%
No. stimuli upper limb	66	71	97
No. stimuli lower limb	67	75	68
No. stimuli mouth	-	-	48
No. stimuli evoking a MEP for upper limb	25	27	45
No. stimuli evoking a MEP for lower limb	15	10	38
No. stimuli evoking a MEP mouth	-	-	10
**DTI tractography**			
CST identification and localization	Lateral displacement. Bundle for hand: anteriorly and bundle for foot: posteriorly	Anterior displacement of hand bundle, posterolateral displacement of the leg bundle	Medial displacement with bundle for mouth markedly shifted anteriorly
FA	Hand: 0.13; foot: 0.16	Hand: 0.15; foot: 0.08	Hand: 0.23; foot: 0.24; mouth: 0.29
DTT (mm)	3	3	4
Fiber integrity	Displaced but intact	Displaced but intact	Displaced but intact

RMT: resting motor threshold, MEP: motor evoked potential, DTI: diffusion tensor imaging, CST: corticospinal tract, FA: fractional anisotropy, DTT: distance tumor tract.

**Table 3:** Intraoperative and postoperative data.

**Intraoperative Data**			
Approach	Trans-sylvian	Trans-temporal	Trans-temporal
IONM	Reduction > 50%	Reduction > 50%	Unchanged
Complications	None	None	None
Pathology	Pilocytic Astrocytoma	Pilocytic Astrocytoma	Pilocytic Astrocytoma
**Postoperative data**			
Complications	MCA stroke	None	None
Residual tumor volume (cm^3^)	0.13 (1.1%)	0.75 (3.9%)	0
Motor status MRC (UL/IL)	M1/M3	M2/M2	M5/M5
Adjuvant therapy	None	None	None
Motor status MRC (UL/IL) at follow-up	M5/M5	M4/M4	M5/M5
Recurrency	None	None	None
Length of follow-up	20	16	12

IONM: intraoperative neuromonitoring, MCA: middle cerebral artery, MRC: motor research council, UL: upper limb, IL: inferior limb.

## Data Availability

All data are available in the text.

## Supplementary Materials

The following supporting information can be downloaded at: https://www.mdpi.com/article/10.3390/brainsci13020234/s1, Table S1: All patients’s data.
