# Supplementary material for: Navigated Transcranial Magnetic Stimulation Motor Mapping and Diffusion Tensor Imaging Tractography for Diencephalic Tumor in Pediatric Patients
Source: Brain Sci. 2023 Jan 30;13(2):234. doi: 10.3390/brainsci13020234 (PMC9954590; doi:10.3390/brainsci13020234)
Supplement: Supplementary file 1 [file brainsci-13-00234-s001.zip › brainsci-2134777-supplementary.pdf]

Table S1 All patients's data

	Patient 1	Patient 2	Patient 3	Patient 4
Preoperative data				
Sex	F	F	F	M
Age	12	13	11	9
Tumour location	Right thalamopeduncular	Right thalamopeduncular	Right thalamic	Left thalamic with temporomesial and frontal invasion
Tumour volume (cm^3)	12.3	19.0	36.7	42.3
Clinical Presentation	Headache and vomiting	Left hemiparesis and VII deficit, hydrocephalus	Headache and vomiting	Headache and vomiting
Motor status MRC grade (UL/IL)	M5/M5	M3/M3	M5/M5	M5/M5
Length of Symotoms (months)	12	7	3	0.2
Antiseizure medications	None	None	None	None
Other	-	NF1	NF1	-
nTMS data				
Duration of the session (min)	73	102	55	
Adverse event	None	None	None	
RMT upper limb	43%	46%	35%	
RMT lower limb	58%	60%	58%	
RMT mouth	Not detected	Not detected	45%	
No. stimuli upper limb	66	71	97	
No. stimuli lower limb	67	75	68	
No. stimuli mouth	-	-	48	
No. stimuli evoking a MEP for upper limb	25	27	45	
No. stimuli evoking a MEP for lower limb	15	10	38	
No. stimuli evoking a MEP mouth	-		10	
DTI tractography				
CST identification and localization	Lateral displacement. Bundle for hand: anteriorly and bundle for foot: posteriorly	Anterior displacement of hand bundle, posterolateral displacement of the leg bundle	Medial displacement with bundle for mouth markedly shifted anteriorly	
FA	Hand:0.13; foot: 0.16	Hand: 0.15; foot: 0.08	Hand: 0.23; foot: 0.24; mouth: 0.29	
DTT (mm)	3	3	4	
Fiber integrity	Displaced but intact	Displaced but intact	Displaced but intact	
Intraoperative data				
Approach	Trans-sylvian	Trans-temporal	Trans-temporal	
IONM	Reduction > 50%	Reduction > 50 %	Unchanged	
Complications	None	None	None	
Pathology	Pilocytic Astrocytoma	Pilocytic Astrocytoma	Pilocytic Astrocytoma	
Postoperative data				
Complications	MCA stroke	None	None	
Residual tumour volume (cm^3)	0.13 (1.1%)	0.75 (3.9%)	0	
Motor status MRC (UL/IL)	M1/M3	M2/M2	M5/M5	
Adjuvant therapy	None	None	None	
Motor status MRC (UL/IL) at follow-up	M5-/M5	M4/M4	M5/M5	

Recurrency	None	None	None	
Length of follow-up	20	16	12	

F: female, M: male, MRC: motor research council, UL: upper limb, IL: inferior limb, RMT: resting motor threshold, MEP: motor evoked potential, DTI: diffusion tensor imaging, CST: corticospinal tract, FA: fractional anisotropy , DTT: distance tumour-tract, IONM: intraoperative neuromonitoring,
